# Evaluation of Myocardial Perfusion Imaging SPECT Parameters and Pharmacologic Stress Test with Adenosine Versus Coronary Angiography Findings: Are They Diagnostically Concordant?

**DOI:** 10.4274/mirt.galenos.2019.47450

**Published:** 2019-06-24

**Authors:** Zekiye Hasbek, Seyit Ahmet Ertürk, Ali Çakmakçılar, İbrahim Gül, Ahmet Yılmaz

**Affiliations:** 1 Cumhuriyet University Faculty of Medicine, Department of Nuclear Medicine, Sivas, Turkey; 2 Cumhuriyet University Faculty of Medicine, Department of Cardiology, Sivas, Turkey

**Keywords:** Myocardial perfusion, SPECT, adenosine, stress test, quantitative parameters

## Abstract

**Objectives:**

In this study our first aim was to evaluate the diagnostic concordance of myocardial perfusion scintigraphy (MPS) by pharmacological stress test with adenosine (APST) with coronary angiography (CAG). The secondary aim of this study was to evaluate the correlation between CAG findings and automated analysis parameters such as left ventricular ejection fraction, summed stress score (SSS), summed rest score, summed difference score (SDS), stress MPS defect percentage ratio (extent) and transient ischemic dilation (TID) obtained by myocardial perfusion imaging single-photon emission computed tomography (SPECT).

**Methods:**

A total of 129 patients (62 male, 67 female, median age: 60.02) undergoing MPS due to suspicion of coronary ischemia who also underwent subsequent CAG in the last year were included in this study, their MPS data and CAG results were compared.

**Results:**

There was no statistically significant diagnostic concordance when visual evaluation of MPS, quantitative MPS parameters and exercise treadmill test (ETT) electrocardiography results were used alone. In fact, diagnostic concordance was higher when automated analysis parameters like TID, SSS and extent values were added to MPS SPECT visual analyses. There was diagnostic concordance in 57.9% of APST patients and 41.7% of ETT patients. There was diagnostic concordance in 75.8% of APST patients and 52.6% of ETT patients who were older than 65 years of age.

**Conclusion:**

In our study, we found that the use of APST during MPS increases diagnostic concordance with CAG. Therefore, we think that it would be appropriate to use APST in women and elderly patients with limited exercise habits. The CAG diagnostic mismatch is far above what it should be when MPS reporting is only done with visual data, and it is not supported by quantitative data such as TID, SSS, SDS and extent.

## Abstract (tr)

**Amaç:**

Bu çalışmada ilk amacımız, adenozin ile farmakolojik stres testinin (AFST), egzersiz treadmill teste (EST) göre koroner anjiyografi (KAG) ile uyumunu araştırmaktır. İkinci amacımız ise, miyokard perfüzyon tek-foton emisyon tomografisi bilgisayarlı tomografi SPECT (MPS) ile elde edilen sol ventrikül ejeksiyon fraksiyon, summed stres skoru (SSS), summed rest skoru, summed difference skoru (SDS), stres MPS defekt yüzde oranı (extent) ve transient iskemik dilatasyon (TID) gibi otomatik analiz parametrelerinin KAG ile uyumunu araştırmaktır.

**Yöntem:**

Çalışmaya son 1 yıl içerisinde koroner arter hastalığı nedeniyle iskemi düşünülerek bölümümüzde MPS yapılan, sonrasında KAG uygulanan 129 hasta (62 erkek, 67 kadın, medyan yaş: 60,02) dahil edildi. MPS verileri ve KAG sonuçları karşılaştırıldı.

**Bulgular:**

Tek başına vizüel değerlendirme, eforlu elektrokardiyografi sonuçları veya sayısal veriler kullanıldığında, KAG ve MPS bulguları arasında istatistiksel olarak anlamlı bir uyum bulunamadı. Oysa MPS vizüel analizine, otomatik analiz parametreleri eklendiğinde KAG ile tanı uyumunun TID, SSS ve extent verileri ile arttığı bulundu. EST yapılan hastaların %41,7’sinde ve AFST yapılan hastaların ise %57,9’unda KAG ile tanı uyumu vardı. Yaşı ≥65 olan hastalardan EST yapılan hastaların %52,6’sında, AFST yapılan hastaların %75,8’inde KAG ile tanı uyumu vardı.

**Sonuç:**

Çalışmamızda MPS sırasında AFST kullanılmasının KAG ile tanı uyumunu artırdığını bulduk. Bu nedenle özellikle efor alışkanlığı kısıtlı olan kadın ve yaşlı hastalarda AFST kullanımının tercih edilmesinin uygun olacağını düşünmekteyiz. MPS raporlama sadece görsel verilerle yapılıp, TID, SSS, SDS ve extent gibi sayısal verilerle desteklenmediğinde KAG uyumsuzluğunun, beklenenin çok üstünde olduğu unutulmamalıdır.

## Introduction

Coronary artery diseases (CAD) is one of the most important causes of mortality and morbidity in the world. Coronary angiography (CAG) is the gold standard in diagnosing CAD. Nevertheless, myocardial perfusion scintigraphy (MPS) using single-photon emission computed tomography (SPECT) with radiopharmaceuticals is widely used for non-invasive diagnosis of obstructive CAD. MPS provides comprehensive information on myocardial perfusion, regional and global left ventricular function that provide incremental diagnostic and prognostic information. MPS evaluates regional myocardial perfusion as well as giving information about functional parameters such as transient ischemic dilation (TID), extent of perfusion defect, etc. (1). A normal stress MPS with adequate stress indicates a very good prognosis, with an annual myocardial infarction or death rate of less than 1-2%. Ischemic perfusion abnormalities usually remain undetected during rest, while stenosis of 50% or more are reliably identified with MPS under maximal myocardial stress. That is why MPS studies are performed with several stress test protocols. Exercise or pharmacological stress augment myocardial blood flow. Although with different mechanisms, myocardial blood flow in coronary vasculature without significant stenosis increases nearly 3-fold with exercise and 3- to 5- fold with vasodilator stressors (2). Exercise (treadmill or bicycle) is the preferred stress modality in patients who can exercise and achieve adequate exercise end-points. The most common mode of stress used in myocardial perfusion imaging is a multi-stage exercise treadmill test (ETT) based on a Bruce or modified Bruce protocol. Pharmacologic stress with adenosine, dobutamine and dipyridamole cause coronary hyperemia and increase myocardial workload allows a successful myocardial perfusion study in patients who cannot perform or tolerate adequate exercise, those with limited heart rate response due to β-blockers or calcium-channel blockers, those with a pacemaker rhythm, Wolff-Parkinson-White syndrome, a transient ventricular pacemaker or with left bundle-branch block. This option is suggested for particular patients in guidelines. The sensitivity and specificity rates of MPS with pharmacological stress test study are reported to be comparable to that of maximal exercise studies, in the range of 85% to 90% (3). A meta-analysis determined the sensitivity and the specificity of adenosine SPECT imaging as 90% and 70%, respectively (4). However, treadmill exercise test is the primarily preferred method for MPS in most nuclear medicine clinics.

In general, SPECT studies are interpreted based on visual assessment of relative tracer uptake images. In clinical practice, imaging equipment, imaging protocols, stress protocols, reconstruction algorithm and filters, the patient’s body habitus, age and gender, artifacts from patient motion, display monitor, the physician’s vision and various other issues affect image evaluation by a nuclear medicine physician. However, automated analysis data from quantitative software tools can be used to assist visual analysis. Quantification is an extremely valuable tool in MPS, as it provides an objective assessment of the parameters under investigation.

Our first aim in this study was to evaluate the concordance of CAG and MPS findings with exercise stress test and pharmacological stress test with adenosine (APST). The second aim in this study was to investigate the correlation between CAG findings and automated analysis parameters such as left ventricular ejection fraction (LVEF), summed stress score (SSS), summed rest score (SRS), summed difference score (SDS), stress myocardial perfusion defect percentage ratio (extent) and TID obtained from MPS SPECT.

## Materials and Methods

### Study Population

This retrospective study was performed in accordance with the Helsinki Declaration. A total of 129 patients (67 female, 62 male, median age: 60.02) who underwent MPS due to suspicion of coronary ischemia and had CAG within the last one year were included in this study. Patients who had motion artifacts in MPS, who had high extra-cardiac activity during MPS, and those who had undergone previous coronary surgery were excluded.

### Stress Protocols (Adenosine and ETT)

Patients were asked to stop taking nitrates for 6 h, calcium-channel blockers for 24 h, and ß-blockers for 48 h prior to ETT. Modified Bruce protocol was used in all patients in ETT. Tc-99m sestamibi was injected when the patient’s heart rate reached 85% of predicted maximum heart rate and exercise was continued for two minutes after the injection.

### Exercise Treadmill Test Procedures

Routine ETT was performed with the use of the standard Bruce protocol. The ETT was continued until the occurrence of marked ST-segment changes, worsening chest pain, sustained ventricular arrhythmias, or excessive fatigue. ST-segment changes, heart rate, and blood pressure measurements were recorded throughout testing. Exertional chest pain or excessive dyspnea was also documented. A normal ETT was defined as the lack of significant ST-segment changes with adequate exercise tolerance. An indeterminate ETT was defined as 0.5 to 1.0 mm of ST-segment changes, exertional chest pain, and/or submaximal exercise tolerance. An abnormal ETT was defined as ≥1 mm of ST-segment change generally occurring in ≥2 leads. The electrocardiography (ECG) was interpreted by site investigators.

This study was approved by the Local Ethics Committee of Sivas Cumhuriyet University (protocol number: 2018-03/06). Consent form was filled out by all participants.

### Stress Testing Procedure with Adenosine

In APST, the adenosine dose was specified as 0.14 mg/kg/min in 100 cc 0.9% NaCl physiological saline solution administered in 6 minutes via intravenous infusion. The Tc-99m sestamibi was injected about halfway into the adenosine infusion (at 3 minutes), when maximal vasodilation and myocardial hyperemia occurred. Heart rate, blood pressure, and a 12-lead electrocardiogram were recorded at baseline and during the study, and for at least 2 minutes after completion of the study.

### Gated SPECT Protocol

All patients underwent the two-day MPS protocol. A dose of ~20-30 mCi Tc-99m sestamibi was injected intravenously for the stress study and ~20 mCi Tc-99m sestamibi was injected for the rest study. All data acquisition was performed with double head SPECT system (DDD-CorCam, Denmark) equipped with a low-energy, high resolution collimator. A protocol consisting of a 64x64 matrix, 30 projections per head, 25-s projections over a 180° circular orbit and 8 frames per cycle was applied, with 140 keV energy photopeak. A rotational arc of 180 degrees was used, beginning at the 45-degree right anterior oblique position and ending at the 45-degree left posterior oblique position with 64 steps in every 3-6 degrees. Image acquisition was done 15-30 minutes after ETT and 45-60 minutes after APST. The gated images were used to assess left ventricle volumes and EF. Gated data acquisition was done with 16 frames per cardiac cycle for the R-R interval length by using the forward-backward gating method.

MPS images were interpreted based on a 17-segment model (5). Images were categorized as either normal or ischemic. Perfusion parameters were derived in an entirely automated fashion using commercially available software program [Cedars-Sinai quantitative perfusion score (QPS) SPECT and quantitative gated SPECT (QGS)]. This program can generate a surface contour even in the apparent absence of perfusion by using smoothness, the iso-contours of the coordinate system, and the geometry of the defect boundaries as constrains. The automatic computations were adjusted manually if left ventricular cavity segmentation was unsuccessful. Visual scan interpretation was performed by at least two experienced readers.

The total score at stress is called SSS that reflects the extent and severity of the abnormality including ischemia and infarction. The difference between the SSS and SRS is called SDS, which reflects a reversible defect.

Semi-quantitative parameters were classified as follows;

A SSS ≤3 was accepted as a normal result, while a score of 4-8 as a mild defect, 9-12 as a moderate defect and >12 as a severe defect (6).

A SDS of 1-3 represented mild ischemia, 4-7 moderate ischemia and >7 severe ischemia (6).

TID indicates a larger left ventricular cavity during stress than rest. TID values were calculated using a commercially available automated program (QPS, Cedars-Sinai). TID more than 1.22 was considered as abnormal (7).

The perfusion defect size correlates with the extent of CAD. Extent indicates perfusion defect area as percent of the mid-myocardial surface area. The perfusion defect extent is calculated as the percentage of the total surface area of the left ventricle, for which test-data are below 3.0 mean absolute deviations (approximately equivalent to 2.5 standard deviations) threshold. Perfusion defect size quantification by percentage size of the left ventricle was classified as (% terms, limits 0-100%): small (0-10%), medium (>10% to 20%), and large (>20%) (6).

LVEF and left ventricular volumes were measured by using QGS. LVEF was also calculated by estimation of end diastolic (EDV) and end systolic volumes (ESV) derived from short axis images [(EDV-ESV) / EDV] x 100. A LVEF <50% was considered as abnormal. CAG data were obtained from cardiac catheterization reports within six months after MPS. Data from MPS and CAG results were compared. If there was an ischemic area in MPS with 50% or more stenosis of coronary arteries in CAG, this result was accepted as concordance in diagnosis. Similarly, if there was not any ischemic area in MPS along with <50% stenosis in coronary arteries in CAG, the result was accepted as concordant diagnosis.

Abbreviations used in the following tables represent;

MPS + (presence of ischemia), MPS - (absence of ischemia), TID + (TID ≥1.22), TID - (TID <1.22), extent (perfusion defect size by percentage size ≥10), extent - (perfusion defect size by percentage size <10), SSS + (SSS ≥4), SSS - (SSS <4), SDS + (SDS ≥4), SDS - (SDS <4).

### Statistical Analysis

Analysis was performed by using SPSS Statistical Software program (SPSS version 23.0, SPSS Inc., Chicago). Diagnostic concordance with CAG was presented by gender, exercise type, MPS visual analysis result, and MPS gated SPECT data (SSS, SDS, extent, TID, LVEF) by using cross tabulations. The chi-square test was used to compare these proportions in different groups. Correlation between automated analysis parameters and stenosis percentage in CAG were evaluated via using Pearson correlation. All continuous variables were described as a mean ± SD. A p value <0.05 was considered as statistically significant.

## Results

One hundred twenty-nine patients were included in the study. Patient demographic features and gated SPECT parameters are summarized in Table 1. There were 67 female patients (51.9%) and 62 male patients (48.1%) in our study. The median patient age was 60.02 years (range: 31-86). There was ≥50% stenosis in a coronary artery in 49 patients (38%), and there was no or <50% stenosis in a coronary artery in 80 patients (62%).

There was diagnostic concordance between MPS and CAG in 57.9% of APST patients and 41.7% of ETT patients (p=0.067). There was diagnostic concordance between MPS and CAG in 67.3% of patients older than 65 years of age and in 36.4% of patients younger than 65 years of age (p=0.001). There was diagnostic concordance between MPS and CAG in 75.8% of APST patients and 52.6% of ETT patients who were older than 65 years of age (p=0.087). There was diagnostic concordance between MPS and CAG in 37.3% of female patients and 61.3% of male patients (p=0.006). Diagnostic concordance between MPS and CAG was significantly higher in APST group than in ETT group, although it was not statistically significant among male patients (p=0.173 for male patients; p=0.046 for female patients) (Table 2).

The mean (standard deviation ±) values of the quantitative MPS parameters of patients with ≥50% coronary artery stenosis in CAG were as follows; SSS: 14.59 (12.1), SDS: 5.86 (5.7), TID: 1.01 (0.14), stress extent 19.06 (16.2), and stress LVEF 55.13 (10). The SSS, SDS, TID and stress extent parameters were statistically significantly higher in patients with ≥50% coronary artery stenosis than in patients with <50% coronary artery stenosis in CAG (p=0.037; 0.029; 0.050; 0.022 and 0.602, respectively.

ETT was performed in 33 female patients and exercise level was inadequate in 10 of those patients (30.3%). Within this group, the ECG result was (+) in 16 patients (48.5%) and was normal in seven patients (21.2%). ETT was performed in 41 male patients and exercise level was inadequate in five of these patients (12.2%). Within this group, ECG result was (+) in 22 patients (53.7%) and was normal in 14 patients (34.1%) (p=0.127).

There was no statistically significant diagnostic concordance between MPS and CAG when visual evaluation of MPS, quantitative MPS parameters and ETT ECG results were used alone. There was 47% diagnostic concordance between MPS and CAG if only visual evaluation of MPS was used. Diagnostic concordance between MPS and CAG was 66.7% when MPS was reported as normal (p=0.194). There was no statistically significant diagnostic concordance between MPS and CAG when SSS, SDS, extent, TID, LVEF and ETT ECG results were used alone (Table 3). In fact, diagnostic concordance was higher when automated analysis parameters like TID, SSS and extent values were added to MPS SPECT visual analysis. Diagnostic concordance between MPS and CAG was 54.1% in MPS + SSS + patients, 60.8% in MPS + extent + patients, and 85.7% in MPS + TID + patients. Moreover, diagnostic concordance between MPS and CAG was 100% in MPS - SSS - patients (only three patients), 83.3% in MPS - extent - patients, and 72.7% in MPS - TID - patients (p=0.021, 0.020 and 0.044, respectively). Although not statistically significant, diagnostic concordance between MPS and CAG was higher in patients with MPS + EF <50% and MPS + SDS + than in patients with MPS + on visual evaluation alone (p=0.055, 0.117, respectively). There was no statistically significant difference in diagnostic concordance between MPS and CAG when ETT results and visual MPS results were evaluated together (p=0.513) (Table 4).

There was low-intermediate or insignificant correlation between CAG and automated analysis parameters (Table 5).

## Discussion

MPS is frequently used for diagnosis and risk stratification in patients with CAD. The anatomical extent of stenosis is poorly correlated with flow reserve and the degree of ischemia. Factors that might impact the functional significance of an anatomical circumferential narrowing include the length, shape, and location of a stenotic lesion. Functional imaging with MPS is often needed to evaluate the clinical significance of a previously known stenosis, particularly in those within the range of 50-70% (8). MPS is a frequently used noninvasive imaging modality for the diagnosis and follow-up of CAD in our country and throughout the world.

Interpretation of MPS images is subjective since several technical and inter-personal features might affect the study. In addition, dilated cardiomyopathy, exercise-induced coronary spasm, mitral valve prolapse, and aortic stenosis have also been associated with various SPECT abnormalities (9).

Although specialists with expertise usually ignore image artifacts that mimic ischemia, false positive findings are still reported frequently because of patient’s clinical situation which can cause image artifacts such as obesity, diaphragm attenuation, breast attenuation and etc. Taking quantitative parameters into consideration along with visual evaluation increases diagnostic accuracy of MPS. Also, artifacts on MPS can cause false positive results. The reproducibility of quantitative analysis of MPS study is higher than visual analysis. Xu et al. (10) reported that quantitative measures of stress, rest and ischemic (stress-rest) defects were significantly more reproducible than visual scores.

Mazzanti et al. (7) determined a sensitivity rate for detection of severe and extensive CAD of 41% by visual analysis as compared to 71% by automatic analysis. Berman et al. (11) found that by perfusion assessment alone, high-risk disease with moderate to severe defects was identified in only 56% of patients visually and in 59% by quantitative evaluation. However, by combining visual perfusion data and nonperfusion variables, especially TID, 83% of patients were identified as high-risk (11). Slomka et al. (12) reported that delayed enhancement MR data and MPS quantitative defect extent percentage showed excellent concordance for detecting the infarct area and its extent. In our study, MPS concordance with CAG was higher than quantitative analysis by visual evaluation alone when the patient was reported as normal (66.7%). However, MPS concordance with CAG was lower than quantitative analysis by visual evaluation alone when the patient was reported as ischemic (47%) (Table 3). In their study with 1148 patients, Chavoshi et al. (13) stated that the incidence of total cardiac events was higher among patients with high SSS and SDS and in those with TID. It is also known that LVEF and left ventricular volumes are important prognostic factors in patients with CAD and left ventricular dysfunction. There is a strong correlation between gated MPI and reference standard measurements of quantitative LVEF, all of which are relatively independent of the isotope, protocol, standard, and algorithm used (14). In a similar manner, TID measured by all the algorithms notwithstanding with effort type is a specific indicator of severe and extensive coronary disease, and TID with positive MPS is accepted as a predictor of poor clinical outcome (15,16). Bourque (17) stated that normal MPS studies with SSS <4 and normal LV function and systolic volumes have a low likelihood of obstructive CAD and a low subsequent event rate in the absence of high-risk comorbidities even with positive TID, and that these patients can be observed with careful follow-up and do not need invasive CAG. However, Abidov et al. (18) indicated that a normal MPS study does not always predict excellent prognosis. TID is an important prognostic factor especially in elderly and diabetic patients. Isolated positivity of TID ratio can be related to diffuse, balanced and severe ischemia (18,19).

In our study, we found that the diagnostic concordance of MPS with CAG increases statistically significantly when TID, SSS and extent ratio (perfusion defect area as percent of the mid-myocardial surface area) were added to visual MPS evaluation. The diagnostic concordance between MPS and CAG was 47% if only visual evaluation of MPS was used. However, the diagnostic concordance was determined as 85.7% in MPS + TID + patients, as 54.1% in MPS + SSS + patients, and as 60.8% in MPS + extent + patients (p<0.05). Diagnostic concordance between MPS and CAG was higher but statistically not significant in patients with MPS + EF <50% and MPS + SDS + than in patients with MPS +, in visual evaluation alone (p=0.055 and 0.117, respectively).

Performing an appropriate and adequate stress test is an important factor that can influence the sensitivity and specifity of an MPS study. According to the EANM guideline, the diagnostic performance of an MPS study is statistically independent of stress agents or modalities (20). A meta-analysis that includes 24 studies and 14.918 patients showed that patients undergoing pharmacologic stress studies are at a higher risk for subsequent cardiac events like myocardial infarction and death from cardiac reasons (21). In contrast, in their prospective study including 266 exercise (bicycle) stress testing and 65 APST, Hochgruber et al. (22) stated that exercise stress but not adenosine stress results in an increase of cardiac wall stress, angina symptoms and ECG changes in patients with reversible ischemic changes on MPS. That is why the absence of these surrogates of myocardial ischemia suggests that adenosine stress does not induce acute myocardial ischemia, but rather displays relative perfusion differences (22). According to American Heart Association data in a study on vasodilator stress in a cohort of 130 women who underwent APST imaging, there was a reported 91% sensitivity and 86% specificity for detecting significant coronary artery stenosis >50% (23). Nevertheless, the same study reported that the sensitivity of MPS with ETT was 78-88% and the specifity was 64-91% (24). In our study, we found that MPS with APST has higher but statistically not significant diagnostic concordance with CAG than MPS with ETT, when all patients were taken into consideration (Table 2). Additionally, we found that the diagnostic concordance with CAG was higher in MPS with APST in both male and female patients than in MPS with ETT (Table 2). This finding was attributed to the low exercise tolerance of patients who have been referred to MPS in our clinics. Interestingly, diagnostic concordance of APST with CAG was higher in patients older than 65 years of age than in ETT (p>0.05). It is the author’s opinion that this situation was related to the fact that APST was the primarily preferred method for elderly patients instead of ETT and that adequate cardiac stress might have been created with APST. In the same patient group, patients did not complete an ETT that can generate adequate cardiac stress. In their WOMEN trial study (The What Is the Optimal Method for Ischemia Evaluation in Women) in low-risk, exercising women, Shaw et al. (25) reported that a diagnostic strategy that uses ETT versus exercise MPS yielded similar two-year post-test outcomes and similar prognosis. In this study, the 85% of predicted maximal heart rate was achieved in 88.4% and 88.1% of patients with MPS and ETT, respectively. However, in our study, 48.5% of female patients performed adequate exercise. Daily life activity and social sports habits may vary depending on country and geographical settlement. Unfortunately, daily sports activities are not routine in our country, especially among women. In addition, exercise capacity decreases significantly in the elderly. According to our study, there was no correlation between ETT and CAG alone. Moreover, ETT did not provide additional contribution in terms of diagnostic concordance with CAG and MPS in our study.

## Conclusion

In our study, we found that the use of APST during MPS increased diagnostic concordance with CAG. Therefore, we think that it would be appropriate to use APST in women and elderly patients with limited exercise habits. However, ETT should be preferred in patients who are thought to be able to perform the test properly and who have a high likelihood of coronary artery stenosis. Evidently, MPS and CAG cannot be expected to comply fully with both physiological (existing collateral circulation, false positive ischemic cardiac pathologies, etc.) and non-physical (due to imaging artifacts, inadequate exercise, etc.) causes. Nevertheless, it should be kept in mind that MPS reporting is based on visual data alone and that CAG diagnostic mismatch is higher than acceptable rates when it is not supported by quantitative data such as TID, SSS, SDS and extent.

## Figures and Tables

**Table 1:**
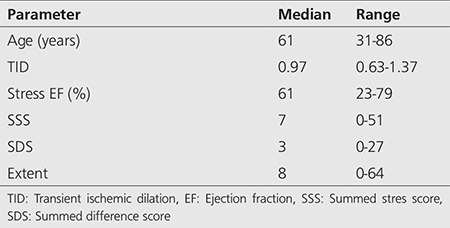
Demographic characteristics and myocardial perfusion scintigraphy gated single-photon emission computed tomography parameters

**Table 2:**
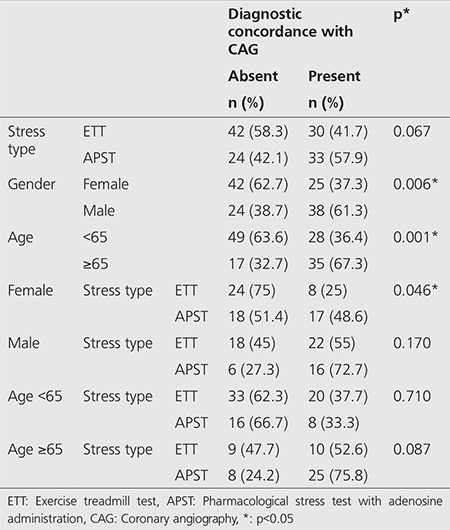
Comparison of stress types, patient gender and age with diagnostic concordance of coronary angiography, p* <0.05

**Table 3:**
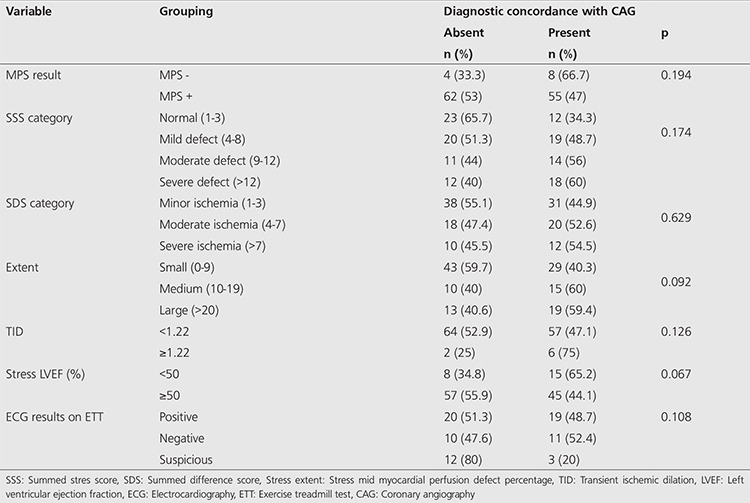
Comparison of automated analysis parameters derived from myocardial perfusion scintigraphy single-photon emission computed tomography and visual evaluation with diagnostic concordance of coronary angiography, *p<0.05

**Table 4:**
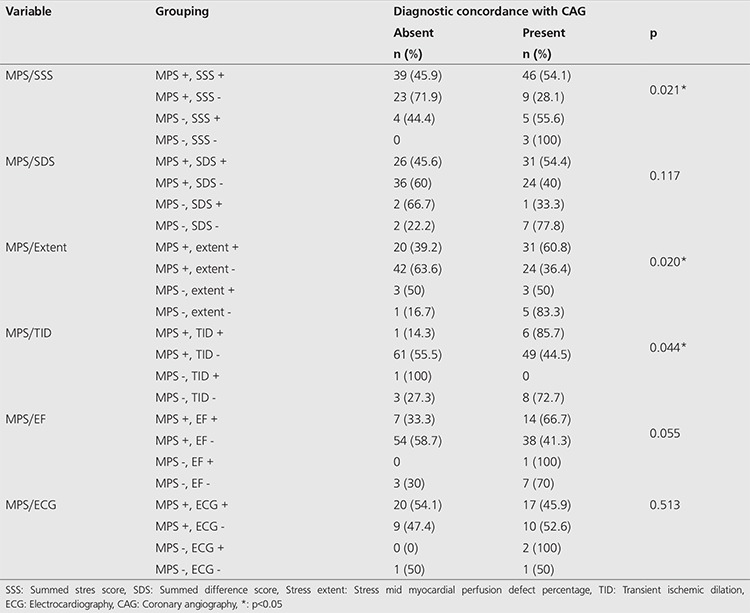
Comparison of automated analysis parameters derived from myocardial perfusion scintigraphy single-photon emission computed tomography and visual evaluation with electrocardiography results in exercise treadmill test with diagnostic concordance of coronary angiography, *p<0.05

**Table 5:**
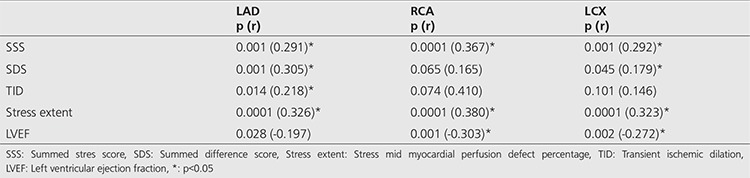
Correlation of automated analysis parameters of myocardial perfusion scintigraphy single-photon emission computed tomography and coronary angiography findings
